# Energetic Optimisation of Foraging Honeybees: Flexible Change of Strategies in Response to Environmental Challenges

**DOI:** 10.1371/journal.pone.0105432

**Published:** 2014-08-27

**Authors:** Anton Stabentheiner, Helmut Kovac

**Affiliations:** Institut für Zoologie, Universität Graz, Graz, Austria; Colorado State University, United States of America

## Abstract

Heterothermic insects like honeybees, foraging in a variable environment, face the challenge of keeping their body temperature high to enable immediate flight and to promote fast exploitation of resources. Because of their small size they have to cope with an enormous heat loss and, therefore, high costs of thermoregulation. This calls for energetic optimisation which may be achieved by different strategies. An ‘economizing’ strategy would be to reduce energetic investment whenever possible, for example by using external heat from the sun for thermoregulation. An ‘investment-guided’ strategy, by contrast, would be to invest additional heat production or external heat gain to optimize physiological parameters like body temperature which promise increased energetic returns. Here we show how honeybees balance these strategies in response to changes of their local microclimate. In a novel approach of simultaneous measurement of respiration and body temperature foragers displayed a flexible strategy of thermoregulatory and energetic management. While foraging in shade on an artificial flower they did not save energy with increasing ambient temperature as expected but acted according to an ‘investment-guided’ strategy, keeping the energy turnover at a high level (∼56–69 mW). This increased thorax temperature and speeded up foraging as ambient temperature increased. Solar heat was invested to increase thorax temperature at low ambient temperature (‘investment-guided’ strategy) but to save energy at high temperature (‘economizing’ strategy), leading to energy savings per stay of ∼18–76% in sunshine. This flexible economic strategy minimized costs of foraging, and optimized energetic efficiency in response to broad variation of environmental conditions.

## Introduction

Honeybees are heterothermic insects which change from the ectothermic to the endothermic state for foraging. They have to keep their body temperature high throughout the entire foraging cycle to stay ready for immediate flight, and to promote fast exploitation of resources [1]–[6]. Endothermy in insects of this small size, however, means enormous efforts to compensate for the high heat loss because of the large surface to volume ratio [7]. This calls for energetic optimisation.

During a foraging trip the challenge is especially high because not only the ambient temperature but also solar radiation may vary in a broad range within a day and during a foraging season [4], [8], [9]. In order to assess the energetic demand of foraging bees under variable ambient temperatures there have been measurements of metabolism in the shade, both at artificial flowers [10]–[14], and during flight [15]–[17]. On many flowers, however, or at water sources honeybees are often not airborne for long periods of time [4], [5], [18], [19]. Since they need not to stay airborne their ability of thermoregulation via regulation of heat production with the thoracic flight muscles is much more pronounced than in flight. Thorax temperature is regulated at different levels depending on several parameters like food quality and demand in the colony [3], [20]–[22]. If weather conditions are fine honeybees prefer foraging in sunshine to get additional heat from solar radiation [5]. Thoracic temperature of foragers in sunshine is usually about 1–3°C higher than in shade [1], [5], [19]. The balancing of body temperature regulation during foraging with the own energetic effort and heat gain from the environment, however, is not well known [6]. The main question is what energetic optimisation strategy honeybee foragers follow. Do they follow general economic principles? Is their energetic and thermoregulatory strategy constant or variable throughout the natural range of ambient temperature variation? An ‘economizing’ strategy of energetic optimisation would be to use external heat gain or high ambient temperatures to minimize foraging costs directly by investing it to save energy via a reduction of the own metabolism. An alternative, more forward directed; ‘investment-guided’ strategy would be to invest heat production and external heat gain to optimize physiological parameters like body temperature which might speed up foraging. Though this would mean an instantaneous increase of costs it might nevertheless optimize foraging indirectly in the longer term by speeding up feeding, pollen gathering and flight, which in turn would decrease costs of a foraging trip. To answer these questions we here present a novel approach of simultaneous measurement of body temperature and energy turnover (from CO_2_ production) of bees foraging sucrose solution from an artificial flower under outdoor conditions, in a broad range of environmental temperature and radiation variation.

## Materials and Methods

### Energetics, thermoregulation and environmental conditions

The experiments were conducted on 11 days in August and September 2004, on 2 days in early October 2005 and on one day in September 2006, between 10∶00 and 16∶00 hours. In order to allow a simultaneous comparison of foraging energetics and thermoregulation in sunshine and shade, 20 individually marked honeybees originating from 15 colonies in an apiary about 10–20 m away were trained to forage 1.5 M sucrose solution ad libitum from inside a brass measurement chamber of ∼7.9 ml inner volume, immersed in a water bath (Julabo F33 HT) outside the laboratory. The chamber lid could be opened and closed quickly to give the bees fast access to an artificial flower inside (for details see [6]). 15 of the 20 bees could be tested both in shade and in sunshine, up to 12 times per radiation condition.

The CO_2_ production was measured with a differential infrared gas analyser (DIRGA; URAS 14, ABB) with a flow-through measurement setup in serial mode according to Stabentheiner et al. [6], operated at a flow rate of 240 ml/min. The loss of measurement gas during chamber opening after the insects’ visits was compensated for by calibrations as described in [6]. Briefly, this procedure compares the washout volumes from the chamber containing certain concentrations of CO_2_ with and without chamber opening.

The ambient air temperature near the foragers was measured by a thermocouple inside the chamber at the air outlet below the bees. The effect of radiation on thermocouple readings was corrected according to [6] if necessary. Solar radiation reaching the bees through the plastic film window of the measurement chamber lid was measured by a photoelectric miniature global radiation sensor in a second chamber beside that containing the artificial flower (FLA613GS/Mini spezial; Ahlborn; see [6]). Convection around the bees was measured with an omnidirectional flow sensor (FV A605 TA, Ahlborn). Environmental data were recorded by ALMEMO data loggers (2690–8 or 2890–9; Ahlborn).

**Figure 1:**
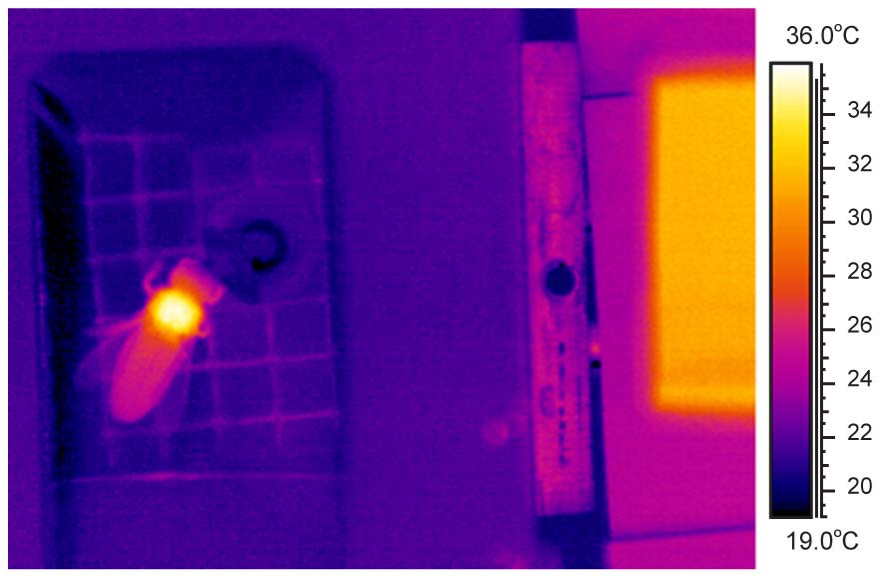
Thermogram of a honeybee foraging sucrose from an artificial flower inside a respiratory measurement chamber. Air inlet is at the bottom of the image, outlet is in the chamber floor right to the bee. The thorax is heated by activation of the flight muscles, part of the heat has reached the head and the abdomen. T_a_ = 21°C. Right-hand rectangle: proprietary infrared reference radiator.

Observation of behaviour and measurement of body surface temperature were done with infrared thermography at a rate of 3–5 Hz (FLIR ThermaCam SC2000 NTS) without behavioural impairment. The infrared camera was calibrated against a peltier-driven reference radiator placed close to the insects (Figure 1; [6]). The attenuation of the infrared radiation by the plastic film was compensated for by covering part of the reference source head with a stripe of the same film. This also minimised errors resulting from ambient reflections via the film surface. In addition, several layers of corrugated cardboard were placed above the infrared (IR) camera. So even in sunshine the lowest cardboard surface temperature resembled the ambient air temperature, which is usually used for correction of reflected ambient radiation. Body surface temperature was calibrated using the cuticular emissivity of the honeybee (0.97; [23]).

### Energy gain

The energy gain from sucrose foraging was determined by training bees to forage from a balance (Mettler Toledo) where their landing and takeoff weight was measured to the nearest 0.1 mg at ambient temperatures of 15–35°C (in shade and in sunshine). The difference was calculated as crop load (in mg). Energy gain from sugar was determined by correcting for density variation due to temperature and using a calorific value of 16.8 kJ/g sucrose [24].

### Data evaluation and statistics

Respiratory data were evaluated in Microsoft Excel and Origin (OriginLab) software. Surface temperatures of head, thorax and abdomen, and of the sucrose solution the bees imbibed, were evaluated at intervals of 3(−5) seconds with ThermaCam Researcher software (FLIR) controlled by a proprietary Excel VBA macro which extracted the stored environmental data automatically from the logger files at the time of thermographic measurement. Statistics and curve fitting was done with Statgraphics (Statpoint Technologies) and Origin software.

All work was conducted according to relevant national and international guidelines.

## Results

### Interrelation of thermoregulation and energetics

**Figure 2:**
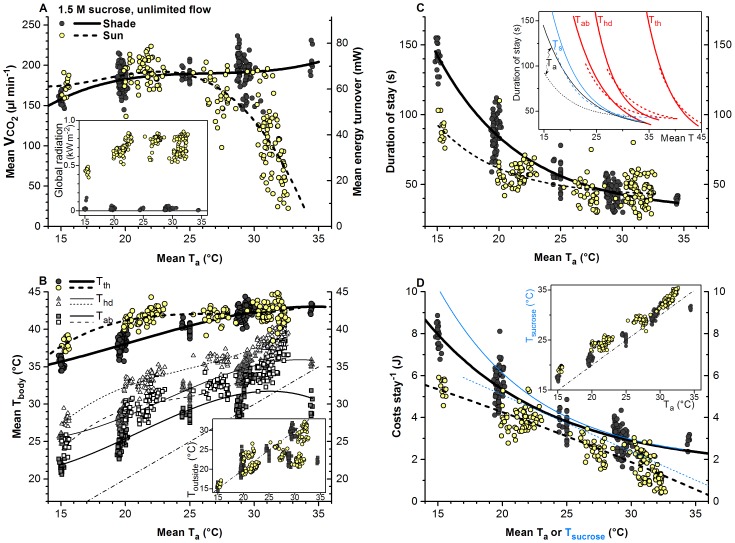
Energetics and thermoregulation of honeybees foraging sucrose in shade (grey/filled symbols) and in sunshine (yellow/open symbols). One symbol represents one mean per stay (N = 217 stays in shade and 183 in sunshine; 20 bees). (A) CO_2_ production rate (VCO_2_), (B) body surface temperature of head (T_hd_), thorax (T_th_) and abdomen (T_ab_), (C) duration of stay, (D) costs per stay, and environmental parameters were measured simultaneously in all individuals. Dashed-dotted line in (B): isoline. T_a_ = ambient air temperature near the bees in the measurement chamber, T_outside_ = temperature in shade outside the measurement chamber, T_s_ = sucrose temperature. Regression lines (all P<<0.0001, ANOVA): (A, B) cubic (y = A+B*x+C*x^2^+ D*x^3^); (C, D) exponential decay (y = A1*e^(−x/t1)+y0)^), for constants and statistics see Table 1. Inserts: if not given, axes labelings as in main graphs; insert in (C): T_a_, T_s_, T_ab_, T_hd_, T_th_ refer to x-axis temperature to be used, respectively.

Once trained properly, the honeybees entered the measurement chamber immediately after arrival and started to drink the sucrose solution from the artificial flower. From a total of 400 visits to the artificial flower 217 measurements were made in shade and 183 in sunshine. The foragers remained endothermic during the whole stay (Figure 1). To our surprise, however, the bees foraging in shade did not decrease energy turnover with increasing ambient temperature (T_a_) but kept it at a high level of ∼56–69 mW on average throughout the investigated range of T_a_ (Figure 2A). This way they were able to increase the thorax surface temperature (T_th_) from ∼35–36°C at T_a_ = 15°C to ∼42–43°C at T_a_ = 30–35°C (Figure 2B).

In sunshine (701 W m^−2^ on average; see insert in Figure 2A) the foragers changed the energetic and thermoregulatory strategy in dependence on T_a_. Below about 25°C they did *not* reduce energy turnover but kept it at a similar level as in shade (∼62–65 mW; Figure 2A). This way T_th_ increased by ∼1–3°C in comparison to the shade (Figure 2B). Above 25°C, by contrast, the bees reduced their energy turnover with increasing T_a_ (∼66–∼6 mW; Figure 2A) in a way that their T_th_ remained at a similarly high level of ∼42–43°C as in shade (Figure 2B). The temperature excess over T_a_ increased in all body parts with decreasing T_a_ (Figure 2B). In the bees exposed to the sun the temperatures of head and abdomen were by about 1–5°C higher than in the bees foraging under shaded conditions. External convection around the bees at our flow rate setting of 240 ml/min amounted to 4.3 cm/s.

### Duration of stay

Both in sunshine and in shade the duration of stay decreased approximately exponential with increasing T_a_ (Figure 2C; Table 1). In shade it changed from ∼140 s at T_a_ = 15°C to ∼40 s at T_a_ = 30–35°C. In sunshine it was considerably lower than in the shade in the lower range of T_a_ (<25°C) but similar at high T_a_ (>25°C), decreasing from ∼90 s at T_a_ = 15°C (∼36% of shade value) to ∼40 s at T_a_ = 30–35°C.

**Table 1:** Constants and statistics for regression functions in Figure 2.

	y = A+B*x+C*x^2^+ D*x^3^		constants				R^2^
	(cubic)		A	B	C	D	
Figure 2A)		shade	−162.1361	39.14352	−1.463	0.01839	0.2762
		sun	455.04686	−51.09818	2.97696	−0.05445	0.7089
Figure 2B)	T_hd_	shade	41.35954	−2.925	0.15998	−0.00231	0.94005
		sun	−28.58677	6.83706	−0.25986	0.00351	0.93368
	T_th_	shade	34.94919	−0.51212	0.04957	–0.0008097	0.88119
		sun	−6.95259	5.44539	−0.20204	0.0025	0.5122
	T_ab_	shade	33.13349	−2.61309	0.16308	−0.00259	0.81108
		sun	−65.26133	11.57206	−0.47612	0.00669	0.87559
	y = A1*e^(−x/t1)+y0)^		**constants**				**R^2^**
	**(exponential decay)**		**y0**	**A1**	**t1**		
Figure 2C)*	duration *vs.* T_a_	shade	30.62809	1026.245	6.76459		0.91215
		sun	41.59258	866.42933	5.30143		0.58617
	duration *vs.* T_sucrose_	shade	33.69157	2791.07346	5.34167		0.90384
	(insert)	sun	41.72591	3531.23814	4.45765		0.57871
	duration *vs.* T_hd_	shade	34.64229	65179.5756	3.95205		0.87412
	(insert)	sun	36.90517	9201.64316	5.35922		0.56006
	duration *vs.* T_th_	shade	15.50437	199780.804	4.77369		0.83793
	(insert)	sun	23.41157	206487.229	4.66765		0.47277
	duration *vs.* T_ab_	shade	24.04419	4112.32539	5.97249		0.82327
	(insert)	sun	40.11864	7351.28361	4.79935		0.47877
Figure 2D)*	costs *vs.* T_a_	shade	1.58628	30.94792	9.49157		0.85673
		sun	22.8322	−14.58653	−82.78026		0.83342
	costs *vs.* T_sucrose_	shade	1.80662	61.13779	7.54753		0.86228
		sun	105668.232	105657.722	−389503.053		0.85918

T_a_ = ambient air temperature; T_hd_, T_th_, T_ab_ = surface temperatures of head, thorax, abdomen. N = 217 in shade and 183 in sun; all regressions significant at P<<0.0001, ANOVA, df = N-4 for cubic and N-3 for exponential decay functions. *: ANOVA linear regression analysis revealed significant differences in slope and intercepts between shade and sunshine at P<0.05, except intercepts for duration *vs.* T_th_ (n.s.).

The duration of stay decreased also approximately exponential with increasing temperature of the sucrose solution (T_s_) but the correlations were somewhat less pronounced than the correlations with T_a_ (see R^2^ values in Table 1). The regressions for shade and sunshine still differed at low T_a_ (<25°C; see insert in Figure 2C). This difference between nonlinear sunshine and shade regressions became much smaller when we correlated the duration of stay with body temperature (insert in Figure 2C). The correlations were best with the temperature of the head (T_hd_; R^2^ = 0.87412 and 0.56006 in shade and in sunshine, respectively), and less pronounced with the temperature of the abdomen (T_ab_; R^2^ = 0.82327 and 0.47877) and of the thorax (T_th_; R^2^ = 0.83793 and 0.47277) (Table 1). ANOVA regression analysis revealed considerable differences between shade and sunshine in most cases (Table 1).

### Costs, gain and efficiency per stay

**Figure 3:**
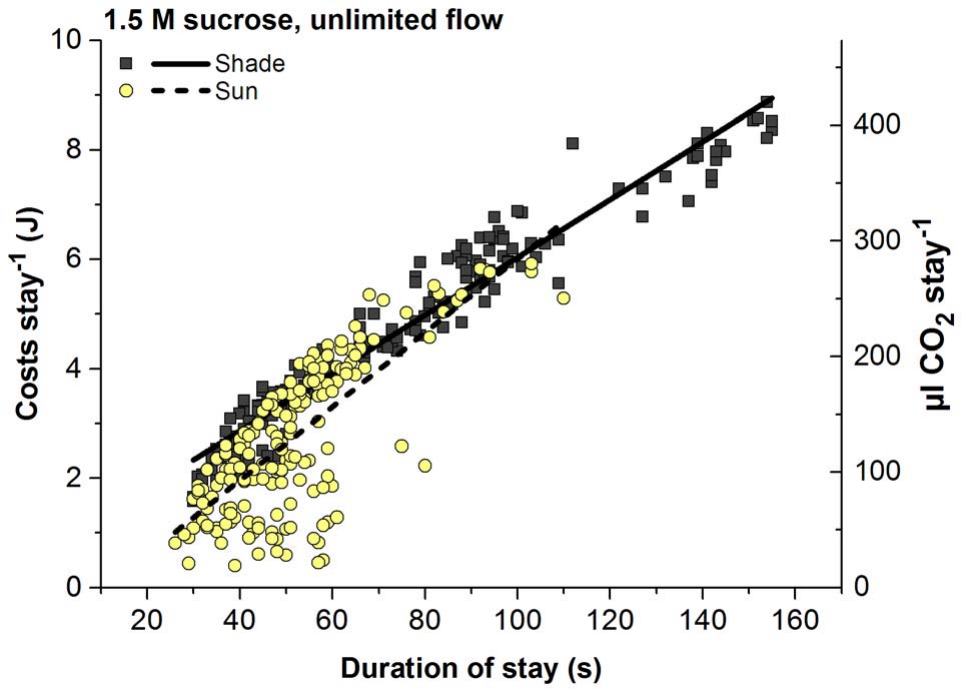
Dependence of costs per stay on duration of stay in shade and in sunshine. Regression line (y = A+B*x) constants A/B: 0.74577/0.05285 (R^2^ = 0.94911) in shade, and −0.74292/0.06753 (R^2^ = 0.55439) in sun (both P<<0.0001, ANOVA; N = 217 in shade and 183 in sun). Regression lines significantly different between shade and sunshine in slope and intercept (P<0.0001, ANOVA).

Energetic costs per stay decreased with increasing T_a_ (Figure 2D; Table 1). Values in shade (as derived from the fitted curves in Figure 2D) amounted to ∼8.0 J at T_a_ = 15°C and ∼2.36 J at T_a_ = 35°C, and in sunshine from ∼5.35 J to ∼0.57 J, respectively. The lower costs in sunshine equal energy savings from external heat gain of 2.65 J, 0.71 J and 1.78 J at a T_a_ of 15, 25 and 35°C if one compares the curves in Figure 2D. This equals savings of ∼33.1%, ∼18.6% and ∼75.7%, respectively. In a similar way as with T_a_ energetic costs per stay decreased with increasing temperature of the sucrose solution (T_s_; thin lines in Figure 2D; Table 1). Figure 3 shows that the energetic costs per stay were a rather straight function of the duration of stay, with similar regressions in sunshine and in shade. In sunshine, however, the range of durations was smaller.

**Figure 4:**
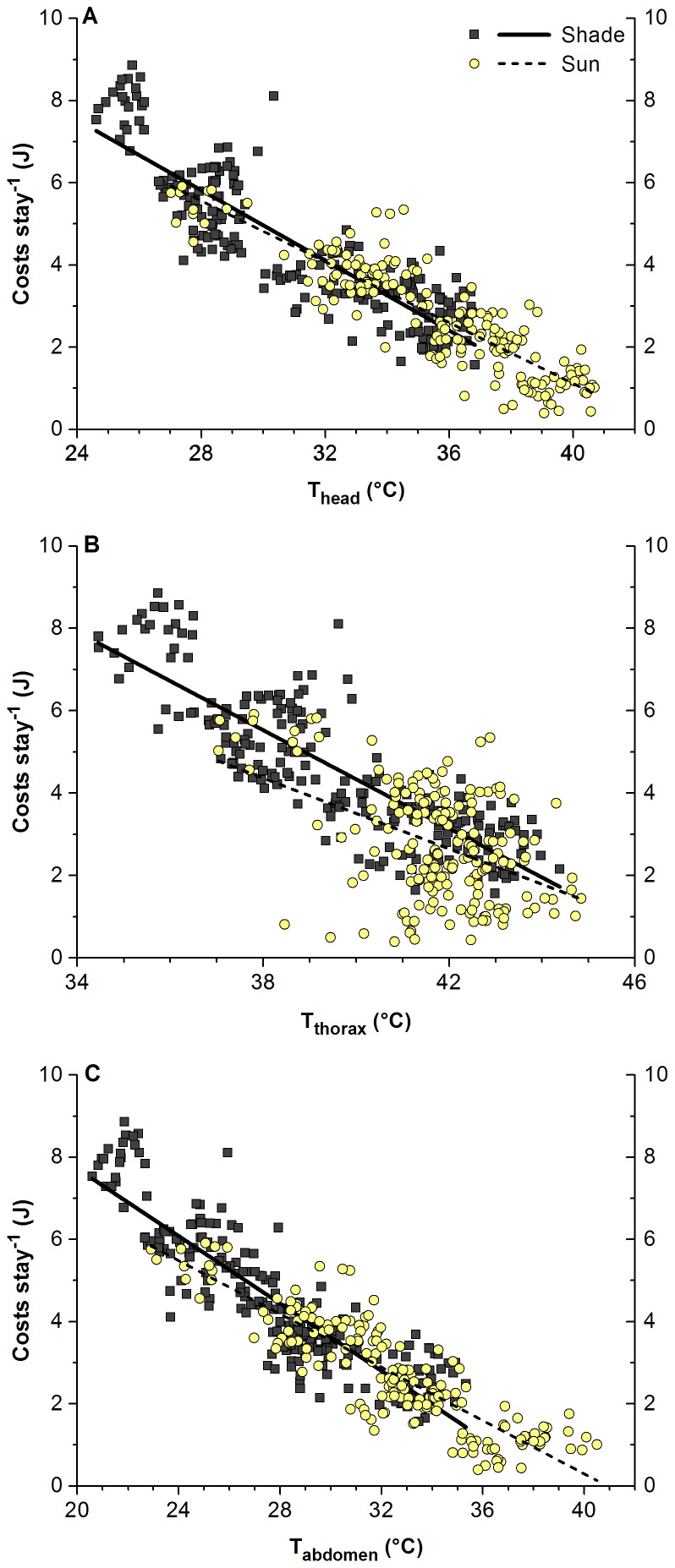
Costs per stay in dependence on temperature of body parts, in shade and in sunshine. Regressions (y = A+B*x; all P<<0.0001, ANOVA; N = 217 in shade and 183 in sun), constants A/B: (A) 17.75233/−0.42623 (R^2^ = 0.76945) in shade, 15.92754/−0.37025 (R^2^ = 0.79089) in sun; (B) 28.17624/−0.59599 (R^2^ = 0.71846) in shade, 20.68964/−0.42959 (R^2^ = 0.20125) in sun; (C) 15.9186/−0.40997 (R^2^ = 0.79066) in shade, 13.26462/−0.32425 (R^2^ = 0.76387) in sun. Regression lines for shade and sunshine significantly different in slope (P<0.05, ANOVA) but not different in intercept for T_head_ (P = 0.4652) and T_abdomen_ (P = 0.6066).

Since body temperature influences duration of stay we analysed the costs per stay in dependence on body part temperature. Costs correlated linearly with the temperatures of all body parts (Figure 4). The best correlations were found with T_head_ (R^2^ = 0.76945 and 0.79089 in shade and in sunshine, respectively) and with T_abdomen_ (R^2^ = 0.79066 and 0.76387). Correlations were less pronounced with T_thorax_ (R^2^ = 0.71848 and 0.20125). The regression lines for shade and sunshine were, though similar, significantly different in slope (P<0.05) but did not differ in intercept for T_head_ (P = 0.4652) and T_abdomen_ (P = 0.6066).

Bees trained to forage 1.5 M sucrose (unlimited flow) from an artificial flower on a balance at ambient temperatures of 15–30°C imbibed a mean amount of sucrose solution of 64.9 mg in shade (SD = 8.38, N = 64) and 64.2 mg in sunshine (SD = 8.69, N = 28) (difference n.s., t test). The imbibed amount was independent of T_a_ in shade (amount (mg) = 65.7741–0.03975*T_a_; R^2^ = −0.01504, P = 0.797) and increased with T_a_ in sunshine (amount (mg) = 45.70473+0.85964*T_a_; R^2^ = 0.37022, P<0.001). Using the mean values we got a mean energetic gain per stay of ∼464 J in shade, and of ∼470 J in sunshine. The measured costs of 8 J down to 0.57 J (Figure 2D) make up only 1.7%, 0.8% and 0.5% of the mean harvested gain in shade, and 1.1%, 0.7% and 0.1% of the mean gain in the sun, at T_a_ = 15, 25 and 35°C, respectively.

Energetic efficiency (gain-costs/costs) (J/J) per stay at our artificial flower was calculated by using the above regressions for imbibed sucrose solution (converted to energy gain) in dependence on T_a_, and the regressions of Figure 2D. Efficiency increased with ambient temperature, in shade from ∼58 to ∼122 and ∼197 (J/J) at T_a_ = 15, 25 and 35°C, and in sunshine from ∼78 to ∼156 and ∼961 (J/J), respectively. Foraging in the sun increased efficiency in the entire range of T_a_ investigated, by ∼35%, ∼28% and ∼387% in comparison to shade, respectively.

## Discussion

### Foraging motivation and energetics

Motivation is an important modulating parameter in foraging honeybees’ thermoregulation and energetics (e.g. [3], [12], [20], [23], [25]). Under our experimental conditions with unlimited flow of 1.5 M sucrose solution, a very high-quality resource, the foraging bees displayed a high energy turnover under most environmental conditions, in shade amounting to about 55–70 mW on average (Figure 2A), which is higher than the 57–60 mW reported by Stabentheiner et al. [6]. Despite foraging 1.5 M sucrose, in shade our bees displayed only a moderately high thorax surface temperature (T_th_) at low to medium T_a_, which was considerably lower than in bees which had foraged a lower concentration (1 M) at a similar distance from the hive [23], and similar to or even somewhat lower than measured in bees foraging only 0.5 M sucrose [3]. We suggest that such differences originate from differences in the bees’ motivational status, which modulates thermoregulation [3], [9], [20], [23], [25] and energy turnover [10]–[13]. In bees foraging from a patch of artificial flowers at limited flow rates, which surely decreases foraging motivation (e.g. [26], [27]), metabolic rate was considerably lower than in the present investigation [12], [13].

### Energy turnover, thermoregulation and environmental variation

It is clear that bees foraging in the shade at low ambient temperature have to be ‘economizing’ in some way because of the high heat loss due to their unfavourable surface to volume ratio [2], [7]. To our surprise the bees foraging under shaded conditions did not follow an ‘economizing’ strategy to reduce the energy turnover with increasing ambient temperature (T_a_) [14], [28] but decided for a graded change to an ‘investment-guided’ strategy: they kept energy turnover rather constant or even increased it (Figure 2A). This resembles independent measurements of oxygen consumption under similar environmental temperature and radiation conditions [6]. Such rather flat energetic curves were also found in flying honeybees [17], in flying carpenter bees (Xylocopa; [29]), and in many other flying insects (e.g. [30]; for more literature see [2]). Conversely, studies have shown that honeybees may decrease metabolic rate and wingbeat frequency during flight at high air temperatures [15], [16]. In flying endothermic moths and honeybees these flat curves are thought to be the result of the limited ability to regulate heat production with the flight muscles because the insects have to stay airborne [2], [17]. In our bees drinking sucrose solution, however, this rather flat energetic curve is surprising, because they *do* have the ability to decrease energy turnover at high T_a_ if necessary as is shown by the values measured in sunshine (Figure 2A). Obviously, the bees changed their energetic strategy more and more to an ‘investment-guided’ one the higher the ambient temperature.

**Figure 5:**
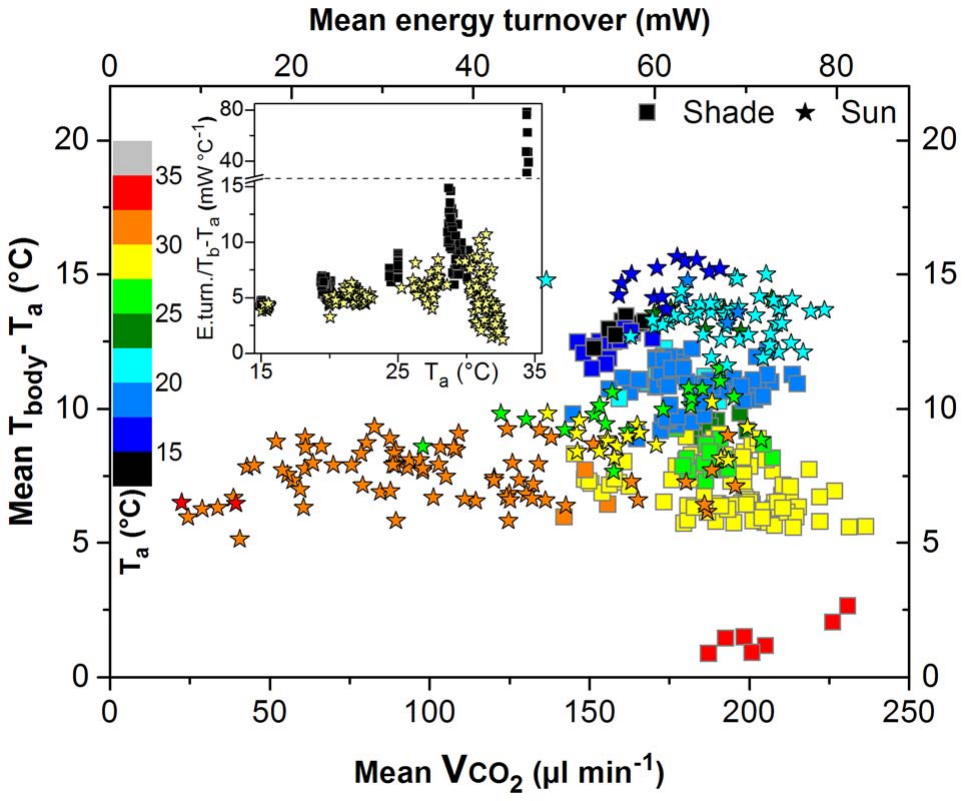
Mean body surface temperature excess over ambient temperature (T_body_–T_a_) per stay, in dependence on mean CO_2_ production rate (VCO_2_) or energy turnover, and T_a_ (colour scale), in shade (squares) and in sunshine (asterisks). Bees foraged 1.5 M sucrose solution from an unlimited flow feeder. Insert: Quotient of energy turnover (E.turn.) and body temperature excess (T_b_–T_a_) in dependence on T_a_. N = 217 in shade and 183 in sun. T_b_ = T_body_ (mean of head, thorax and abdomen).

Though in shade the CO_2_ production was nearly independent of T_a_ (Figure 2A) and T_th_ decreased with decreasing T_a_ (Figure 2B), the bees managed to thermoregulate to some extent: the thoracic temperature excess over T_a_ (T_th_–T_a_) increased with decreasing T_a_ (Figure 2B). One might assume that body temperature and energy turnover are in a simple interrelationship. In honeybees heating their thorax up in flight preparation Goller and Esch [31] reported a straight increase of T_th_–T_a_ with the metabolic turnover. Our simultaneous measurements of body temperature and CO_2_ production uncovered a considerable variability with no simple relationship (Figure 5; compare [32]). The dependence of T_th_-T_a_ on the energy turnover necessary to reach a certain excess temperature changed with T_a_ both in shade and in sunshine (Figure 5). Therefore, the bees foraging in the shade must have regulated body temperature in reaction to changes of T_a_ primarily not by regulation of heat production but by regulation of heat loss. The decrease of a ‘conductance’ estimate in shade with decreasing T_a_ (energy turnover per degree body temperature difference to T_a_; insert in Figure 5, dark symbols) supports this interpretation. We suggest that the bees did not have much regulatory ability left at the lowest T_a_ but utilized this ability at higher T_a_. The low and constant external convection of 4.3 cm/s around the bees was probably not a major source of heat loss. Regulation of heat loss via cooling of the head by regurgitated fluid droplets at high T_a_ [33]–[35] was probably also not of much importance because the bees imbibing the sucrose solution had wet mouthparts anyway, suggesting a rather constant cooling effect. The two remaining pathways of heat loss regulation are the heat transport to the abdomen, which is not so much pronounced in honeybees [2], and respiration. Regulation of respiratory heat loss might be accomplished by modulation of ventilation frequency for example.

Our investigation shows for the first time that honeybees follow a flexible strategy concerning the use of solar radiation. Instead of reducing energy turnover at low T_a_ they invested solar heat to increase the temperature of all body parts considerably (Figure 2, ‘investment-guided’ strategy). At high T_a_, by contrast, they decreased their energy turnover in the sun (‘economizing’ strategy), probably to prevent overheating. Though honeybees are rather heat tolerant insects [36], [37] a further increase of body temperature might nevertheless have been unfavourable in the long term [37].

### Body temperature and suction speed

The energy turnover measured in the present study was considerably higher than the turnover measured in most studies of agitated or hovering flight, where means amounted to about 38–63 mW [15]–[17], [38], [39]. This is surprising in so far as in our experiments the bees had not to lift their weight in flight. What is the purpose of this extreme investment? Figure 2 shows that the foragers used both the high energy turnover and solar heat to increase body temperature in a flexible manner, which led to the concurrent exponential decrease of the duration of stay (increase of suction speed) with increasing T_a_ (Figure 2C; [40]). The function of the musculature involved in ingestion of fluids (‘suction pump’, cibarium with associated structures) is suggested to be strongly dependent on body temperature [19].

The extraordinarily high values of T_th_ at high T_a_ (T_th_ = 40–44.5°C; Figure 2B) were not primarily a means to achieve maximum lift for immediate readiness for takeoff. Coelho [41] has shown that bees reach their maximum achievable lift already with a T_th_ of ∼39°C. A higher T_th_ has an inhibitory effect on flight muscle performance. In bees foraging from flowers where they often must remain prepared for immediate commencement of flight, T_th_ usually remains below 40°C [1], [4], [5]. On water sources, on the other hand, where the bees can speed up foraging with a higher body temperature like at our artificial flower (unlimited flow of resources in both cases), a T_th_ in sunshine higher than 40°C was nearly as common as in our experiments [19].

### Costs, gain and efficiency

The balance between energy investment and energy gain is crucial in foraging insects. With their flexible strategy the bees’ costs per stay decreased considerably with increasing T_a_ both in shade and in sunshine (Figure 2D). The main parameter determining the costs per stay under our experimental conditions of unlimited sucrose flow was time: costs increased linearly with the duration of stay (Figure 3). The linear decrease of the costs with body temperature (Figure 4) supports the hypothesis that the body temperature, especially that of head and abdomen, was the main factor determining efficiency of foraging [19]. The temperature of the thorax is of course also important because the heat produced there is transferred to the head and to the abdomen in part. It is noteworthy that the costs for sunny conditions are closely in the trend of those from the shade, with identical intercepts for head and abdomen temperature (Figure 4). This again emphasizes the interpretation that the bees use solar radiation in a flexible way to optimize body temperature for the purpose of optimisation of foraging efficiency.

Beside the body temperature, also the temperature of the imbibed food (of nectar or water) influences honeybee foraging [19], [42]–[44]. On flowers and cold water sources bees always prefer the warmer or sunny patches over colder or shaded ones [5], [19]. Beside the direct effect of the sun on body temperature [5], [19] warmer nectar or water will cool the mouthparts and suction pump less and this way probably contributes to an improved function. This way the bees can make more foraging trips per time interval, which increases the harvested amount of sugar (energy) per day.

In any case, foraging in the sun enabled the foragers to reduce the energetic costs per stay considerably, by about 19% to 76% (compare Figure 2D). At low T_a_ this was achieved by an increase of body temperature and the resulting increase of the suction speed. This points to an ‘investment-guided’ strategy under these conditions which promises additional gain in return. This maximising of returns would not be accessible with an energy-saving (‘economizing’) strategy. At high T_a_, by contrast, it was the reduction of the energy turnover which made these savings possible. This equals an ‘economizing’ strategy.

It has to be kept in mind, however, that experiments with unlimited flow of highly concentrated sugar solution provide the bees with an enormous gain per unit of time [45]. The costs of 8 J down to 0.57 J (Figure 2D) make up a rather small fraction of the energy gain (1.7% to 0.5% in shade and only 1.3% to 0.1% in sunshine). The relation of gain to costs will be much less favourable under conditions of low (limited) nectar flow [12]. The high turnover observed not only in shade but even in sunshine is not a waste of energy but an investment which maximizes the profitability of foraging by optimizing energetic efficiency (gain-costs/costs) [45]–[50]. Foraging in the sun increased efficiency in the entire range of T_a_ investigated (by ∼28% to ∼387%). However, this was not accomplished by a constant but by a *flexible* physiological and behavioural strategy of own energetic investment and use of external (solar) heat.

We conclude that foraging honeybees follow a flexible economic strategy. They change between an ‘economizing’ or an ‘investment-guided’ strategy on demand. This optimizes body temperature in a graded manner in reaction to environmental variation, and this way maximizes intake rate of the colony.
